# Weekly platinum-based chemotherapy versus 3-weekly platinum-based chemotherapy for newly diagnosed ovarian cancer (ICON8): quality-of-life results of a phase 3, randomised, controlled trial

**DOI:** 10.1016/S1470-2045(20)30218-7

**Published:** 2020-07

**Authors:** Sarah P Blagden, Adrian D Cook, Christopher Poole, Lesley Howells, Ian A McNeish, Andrew Dean, Jae-Weon Kim, Dearbhaile M O'Donnell, Jane Hook, Elizabeth C James, Ian R White, Timothy Perren, Rosemary Lord, Graham Dark, Helena M Earl, Marcia Hall, Richard Kaplan, Jonathan A Ledermann, Andrew R Clamp

**Affiliations:** a Department of Oncology, University of Oxford, Oxford, UK; b Medical Research Council Clinical Trials Unit, Institute of Clinical Trials and Methodology, University College London, London, UK; c Department of Oncology, University Hospital Coventry, Coventry, UK; d Maggie Keswick Jencks Cancer Caring Centres Trust, London, UK; e Ovarian Cancer Action Research Centre, Department of Surgery and Cancer, Imperial College London, London, UK; f Oncology Department, St John of God Subiaco Hospital, Perth, WA, Australia; g Department of Obstetrics and Gynaecology, Seoul National University Hospital, Seoul, South Korea; h Gynaecology Subgroup, Cancer Trials Ireland, Dublin, Ireland; i St James's University Hospital, Leeds, UK; j Department of Oncology, Clatterbridge Cancer Centre, Birkenhead, UK; k Department of Oncology, Newcastle University, Newcastle, UK; l NIHR Cambridge Biomedical Research Centre, Cambridge, UK; m Department of Medical Oncology, Mount Vernon Cancer Centre, Northwood, UK; n UCL Cancer Centre Institute, University College London, London, UK; o University College Hospital, London, UK; p Department of Medical Oncology, The Christie NHS Foundation Trust, Manchester, UK; q University of Manchester, Manchester, UK

## Abstract

**Background:**

The ICON8 study reported no significant improvement in progression-free survival (a primary endpoint) with weekly chemotherapy compared with standard 3-weekly treatment among patients with epithelial ovarian cancer. All ICON8 patients were eligible to take part in the accompanying health-related quality-of-life study, which measured the effect of treatment on self-reported wellbeing, reported here.

**Methods:**

In this open-label, randomised, controlled, phase 3, three-arm, Gynecologic Cancer Intergroup (GCIG) trial done at 117 hospital sites in the UK, Australia, New Zealand, Mexico, South Korea, and Republic of Ireland, women (aged at least 18 years) with newly diagnosed, histologically confirmed International Federation of Gynecology and Obstetrics stage IC–IV ovarian cancer and an Eastern Cooperative Oncology Group performance status of 0–2 were randomly assigned (1:1:1) centrally using minimisation to group 1 (intravenous carboplatin area under the curve [AUC]5 or AUC6 and 175 mg/m^2^ intravenous paclitaxel every 3 weeks), group 2 (carboplatin AUC5 or AUC6 every 3 weeks and 80 mg/m^2^ paclitaxel weekly), or group 3 (carboplatin AUC2 weekly and 80 mg/m^2^ paclitaxel weekly). Randomisation was stratified by GCIG group, disease stage, and outcome and timing of surgery. Patients and clinicians were not masked to treatment assignment. Patients underwent immediate or delayed primary surgery according to clinicians' choice. Patients were asked to complete European Organisation for Research and Treatment of Cancer QLQ-C30 and QLQ-OV28 questionnaires at enrolment, before each chemotherapy cycle, then 6-weekly up to 9 months, 3-monthly up to 2 years, and 6-monthly up to 5 years. Quality of life was a prespecified secondary outcome of the ICON8 study. Within the quality-of-life study, the co-primary endpoints were QLQ-C30 global health score at 9 months (cross-sectional analysis) and mean QLQ-C30 global health score from randomisation to 9 months (longitudinal analysis). Data analyses were done on an intention-to-treat basis. The trial is registered on ClinicalTrials.gov, NCT01654146 and ISRCTN Registry, ISRCTN10356387, and is currently in long-term follow up.

**Findings:**

Between June 6, 2011, and Nov 28, 2014, 1566 patients were recruited into ICON8 (522 were included in group 1, 523 in group 2, and 521 in group 3). Baseline quality-of-life questionnaires were completed by 1438 (92%) of 1566 patients and 9-month questionnaires by 882 (69%) of 1280 patients. We observed no significant difference in global health score at 9 months (cross-sectional analysis) between study groups (group 2 *vs* group 1, difference in mean score 2·3, 95% CI −0·4 to 4·9, p=0·095; group 3 *vs* group 1, −0·8, −3·8 to 2·2, p=0·61). Using longitudinal analysis, we found lower global health scores for those receiving weekly paclitaxel than for those receiving 3-weekly chemotherapy (group 2 *vs* group 1, mean difference −1·8, 95% CI −3·6 to −0·1, p=0·043; group 3 *vs* group 1, −2·9, −4·7 to −1·1, p=0·0018).

**Interpretation:**

We found no evidence of a difference in global quality of life between treatment groups at 9 months; however, patients receiving weekly treatment reported lower mean quality of life across the 9-month period after randomisation. Taken together with the lack of progression-free survival benefit, these findings do not support routine use of weekly paclitaxel-containing regimens in the management of newly diagnosed ovarian cancer.

**Funding:**

Cancer Research UK, Medical Research Council, Health Research Board Ireland, Irish Cancer Society, and Cancer Australia.

## Introduction

ICON8 was an international, randomised, controlled, Gynecologic Cancer Intergroup (GCIG), phase 3 study to evaluate weekly dose-dense paclitaxel-containing chemotherapy compared with standard 3-weekly chemotherapy (carboplatin plus paclitaxel) in patients with newly-diagnosed ovarian cancer. The study was prompted by the phase 3 JGOG-3016 trial in which weekly paclitaxel (80 mg/m^2^) in combination with 3-weekly carboplatin (area under the curve[AUC]6) was found to confer improved progression-free survival and overall survival when compared with standard treatment in Japanese patients with advanced ovarian cancer.^1^ As there is increasing evidence of pharmacogenomic distinctions between Asian and white populations, ICON8 was designed to explore whether the survival advantage observed in JGOG-3016 could also be observed in a mostly European population with ovarian cancer.^2^, ^3^ Results from ICON8 have shown that weekly, dose-dense paclitaxel-containing chemotherapy conferred no progression-free survival advantage when compared with standard 3-weekly treatment.^4^ As a secondary endpoint of the main study, the effect on health-related quality of life (referred to as quality of life throughout this Article) was assessed during treatment and in follow-up. Here we report the quality-of-life results from the ICON8 study.

**Research in context**

**Evidence before this study**

We searched PubMed, MEDLINE, life science journals, and online books, including non-English language publications, from database inception until April 15, 2018, for prospective cohort and randomised trials using the search terms “ovarian cancer” and “weekly paclitaxel” and “carboplatin”. We excluded reviews and studies of other chemotherapy combinations or in different disease contexts. We selected trials in patients with primary ovarian cancer where health-related quality of life assessment was done. Our search identified three studies: JGOG-3016, GOG-0262, and MITO-7.

The Japanese JGOG-3016 study reported improved survival outcomes with no detriment to quality of life in patients with newly diagnosed ovarian cancer for weekly, dose-dense, paclitaxel and 3-weekly carboplatin, compared with standard 3-weekly carboplatin and paclitaxel. The GOG-0262 study compared similar dose schedules, but most patients also received bevacizumab, and the study reported no progression-free survival benefit and poorer quality of life with weekly treatment compared with 3-weekly treatment. The MITO-7 study compared weekly carboplatin plus weekly paclitaxel with 3-weekly carboplatin plus 3-weekly paclitaxel and reported improved quality of life with weekly treatment over the 9-week evaluation period. The evidence from these three studies was that weekly paclitaxel-containing treatment was superior to, or at best equivalent to, standard 3-weekly treatment with little quality-of-life detriment to patients.

**Added value of this study**

To our knowledge, ICON8 is the largest study to date of weekly treatment for primary ovarian cancer. The study showed no progression-free survival advantage for patients receiving dose-dense paclitaxel-containing chemotherapy compared with 3-weekly carboplatin and paclitaxel. The quality-of-life results show that, although the global quality of life of patients was similar between the three treatment groups at 9 months, those receiving dose-dense paclitaxel had poorer quality of life during chemotherapy treatment, with more severe peripheral neuropathy that lasted for up to 18 months.

Patients could have either immediate primary debulking surgery followed by chemotherapy or upfront chemotherapy that was interrupted by delayed primary surgery; patients who had delayed surgery reported less detriment to quality of life with weekly treatment than did patients with immediate surgery.

**Implications of all the available evidence**

The contrasting results of ICON8 and JGOG-0316 support a differential response to dose-dense paclitaxel between Asian and white patients. With no progression-free survival benefit and poorer quality of life, the ICON8 results do not support the general use of weekly treatment among a primarily European population. However, by allowing more precise dose modulation and symptom management, weekly treatment might still be appropriate for some patients. The observed difference between patients who received immediate versus delayed surgery highlights the importance of prognosis in future studies.

## Methods

### Study design and participants

ICON8 was an open-label, randomised, controlled, phase 3, three-arm trial done at 117 hospital sites in the UK, Australia, New Zealand, Mexico, South Korea, and Republic of Ireland (appendix pp 1–3). Detailed methods for ICON8 have been previously reported.^4^ Eligible patients were aged 18 years or older and had histologically confirmed, newly diagnosed high-risk International Federation of Gynecology and Obstetrics (FIGO) stage IC-IIA or any advanced (FIGO stage IIB-IV) epithelial ovarian, primary peritoneal, or fallopian tube carcinoma (collectively termed ovarian cancer). High-risk histologies included high-grade serous carcinoma, clear cell carcinoma, or other poorly differentiated and grade III subtypes. Other inclusion criteria were an Eastern Cooperative Oncology Group (ECOG) performance status of 0–2; life expectancy longer than 12 weeks; adequate haematological, renal, and hepatic function; and able to start chemotherapy within 8 weeks after immediate primary debulking surgery. Patients were also eligible for the trial if there was no plan for surgery or planned delayed primary surgery after neoadjuvant chemotherapy. Patients had not received previous systemic therapy for ovarian cancer and were not scheduled to receive maintenance treatment after completion of protocol therapy. All patients gave written informed consent to join the trial.

Exclusion criteria included previous malignancy within 5 years, previous or synchronous early-stage endometrial cancer, evidence of brain metastasis, and pre-existing sensory or motor neuropathy of grade 2 or above.

In the UK, ethical approval was granted by the London–Chelsea research ethics committee. The trial also received ethical approval from appropriate national or local institutional review boards in other jurisdictions. The protocol can be found online.

### Randomisation and masking

Patients were randomly assigned (1:1:1) to standard three-weekly carboplatin and paclitaxel (group 1), three-weekly carboplatin and weekly dose-dense paclitaxel (group 2), or weekly carboplatin and weekly dose-dense paclitaxel (group 3; appendix p 4). Patients were randomly assigned with the Medical Research Council Clinical Trials Unit University College London randomisation telephone service; the method of minimisation was used with stratification factors of GCIG group, disease stage (FIGO stage IC high grade serous, clear cell, or grade III carcinoma; stage IIA high grade serous, clear cell, or grade III carcinoma; stage IIB; stage IIC; stage IIIA; stage IIIB; stage IIIC; stage IV), and outcome and timing of surgery (immediate surgery plus FIGO stage IC–III with no visible residual disease; immediate surgery plus FIGO stage IC–III with residual disease ≤1 cm; immediate surgery plus FIGO stage IV or IC–III with residual disease >1 cm; no surgery currently planned; or delayed primary surgery planned). Patients and clinicians were not masked to their allocated group.

### Procedures

Patients entering the trial could have upfront debulking surgery before starting chemotherapy, referred to as immediate primary surgery, delayed primary surgery after at least three cycles of neoadjuvant chemotherapy, or no planned surgery. As with standard practice, the choice between immediate and delayed surgery was decided by patients' local gynaecological oncology teams at multidisciplinary meetings. All participants started chemotherapy within 2 weeks of randomisation. Patients in group 1 received carboplatin area under the curve [AUC]5 or AUC6 by intravenous infusion over 30–60 min and paclitaxel 175 mg/m^2^ by intravenous infusion for 3 h on day 1 of a 21-day cycle for six cycles; patients in group 2 received carboplatin as in group 1 and dose-fractionated paclitaxel 80 mg/m^2^ by intravenous infusion for 1 h on days 1, 8, and 15 of a 21-day cycle for six cycles; and patients in group 3 received carboplatin AUC2 by intravenous infusion for 30–60 min on days 1, 8, and 15 and paclitaxel 80 mg/m^2^ by intravenous infusion for 1 h on days 1, 8, and 15 of a 21-day cycle for six cycles. Additional details regarding treatment have been published.^4^ Protocol-defined dose alterations (delay, reduction, or omission) were allowed for haematological and other toxic effects if deemed clinically necessary, as described previously.^4^

All patients were included in the quality of life study and invited to complete European Organisation for Research and Treatment of Cancer QLQ-C30 and QLQ-OV28 questionnaires to provide a subjective measure of their quality of life over the preceding 7 days. The QLQ-C30 contains 30 items, including a global health status score, five function scales (physical, role, emotional, cognitive, and social) and nine symptom scales or items (fatigue, nausea or vomiting, pain, dyspnoea, insomnia, appetite loss, constipation, diarrhoea, and financial difficulties).^5^ The QLQ-OV28 contains 28 items relevant to ovarian cancer, including abdominal or gastrointestinal symptoms, peripheral neuropathy, chemotherapy side-effects, hormonal or menopausal symptoms, body image, attitude to disease or treatment, and sexual functioning.^6^ For global health status and function scales, higher scores indicate better function (improved quality of life) but for symptom scales, higher scores indicate greater symptoms (poorer quality of life). The QLQ-C30 and QLQ-OV28 questionnaires have undergone extensive psychometric validation and multiple translations and are acceptable to patients.^5^, ^6^, ^7^ We interpreted a change in global health score over time of more than 5 points as being clinically significant following the methodology of Cocks and King, who defined a change in the score of less than 5 as clinically trivial.^8^, ^9^, ^10^

**Figure 1:**
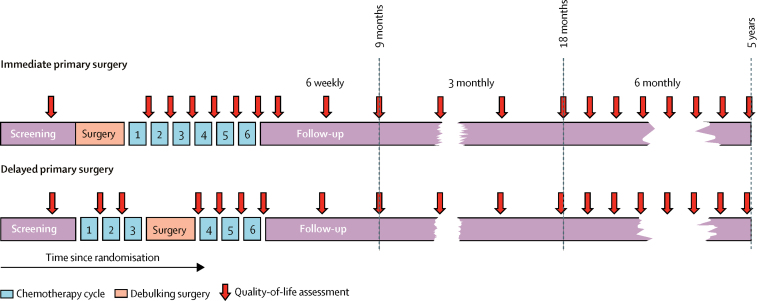
Timing of quality-of-life questionnaires

QLQ-C30 and QLQ-OV28 questionnaires were completed during outpatient attendances at day 1 of each chemotherapy cycle and during follow-up visits 6-weekly until 9 months from randomisation, 3-monthly to 2 years from randomisation, and then 6-monthly for up to 5 years from randomisation (figure 1). Where possible, the questionnaires were also completed at 6-month intervals after disease progression. Questionnaires were given to patients in paper format and completed without conferring with others before treatment administration or medical consultations. Centres were asked to report reasons for any missing questionnaires.

### Outcomes

The trial had co-primary endpoints of progression-free survival and overall survival, which have been previously reported.^4^ A secondary outcome, quality of life, is reported here. The quality-of-life study comprised two co-primary endpoints: cross-sectional analysis of QLQ-C30 global health score 9 months after randomisation and longitudinal analysis of QLQ-C30 global health score from randomisation to 9 months. Secondary quality-of-life endpoints were defined from four clinically relevant function and symptom scores, as follows: QLQ-C30 emotional function, QLQ-C30 social function, QLQ-C30 fatigue, and QLQ-OV28 peripheral neuropathy. These endpoints were analysed cross-sectionally at 9 months and longitudinally from randomisation to 9 months. Analyses of all other function and symptom scores up to 18 months after randomisation were treated as exploratory.

### Statistical analysis

The sample size of ICON8 was determined to detect a hazard ratio of 0·75 in progression-free survival between groups 1 and 2, and groups 1 and 3, with two-sided 2·5% significance and 90% power. For quality of life, a retrospective power calculation showed that the study had 90% power to detect a difference between groups of 5 points in global quality of life using the SD observed in group 1. The quality-of-life study adhered to the null hypothesis of the main ICON8 study—no expected difference in quality of life between the three randomised treatment groups. A quality-of-life expert panel (comprised of LH, CP, SPB, and RK), invited by the trial management group, convened on June 9, 2016, to define the primary, secondary, and exploratory endpoints.

We compared each weekly treatment group with the 3-weekly group, following the main analysis of clinical endpoints (group 2 *vs* group 1, and group 3 *vs* group 1). To adjust for two comparisons against the same control group, we used a two-sided significance level of p=0·025 to judge statistical significance in primary and secondary analyses. Descriptive data are presented for all other validated subscales of the QLQ-C30 and QLQ-OV28.

For cross-sectional comparisons of quality-of-life outcomes at 9 months, we used analysis of covariance adjusted for baseline score, hence omitting data from the chemotherapy period. Longitudinal analyses used all data collected from baseline to 9 months; we estimated scores at scheduled data collection points from a mixed effects regression model with a time–treatment interaction, unstructured covariance, and patient level random effects. We then calculated the AUC for each treatment group from the fitted model. Results are presented as mean quality of life (ie, mean height of the curve) over the 9-month period. The 9-month timepoint was chosen as it represents a period of good quality of life for most patients—few will have had disease progression, and most will have completed treatment some months earlier. It is also the timepoint in ICON8 at which follow-up, and hence data collection, changed from 6-weekly to 3-monthly. The co-primary endpoints of cross-sectional and longitudinal changes in global health score are complementary—longitudinal analysis compares patient experience across the whole 9-month period and cross-sectional analysis compares scores at the 9-month timepoint. Thus, longitudinal analysis makes better use of the data and is the preferred method, whereas cross-sectional analysis provides a post-treatment snapshot.

For patients who had neo-adjuvant chemotherapy and underwent delayed primary surgery, chemotherapy cycles 4 to 6 and the end of treatment visit were around 4 weeks later than equivalent visits in the immediate surgery group. Therefore, quality-of-life data were ordered by visit number rather than date.

Three post-hoc analyses were done: analysis of the primary and secondary outcomes separately for immediate surgery and delayed surgery patients (including a comparison of baseline quality of life scores), a comparison of peripheral neuropathy scores from self-reported quality-of-life data with neuropathy adverse event data from clinicians (according to Common Terminology Criteria for Adverse Events version 4.0),^4^ and analysis of self-reported peripheral neuropathy scores 18 months after randomisation.

We assessed the potential effect of missing data on the co-primary outcome using imputation to model the following scenarios: scenario 1, a global score of 0 was imputed for patients who died within 9 months of enrolment; scenarios 2–4, all patients alive and without progression at 9 months but missing quality-of-life data were assigned a score, starting respectively with the mean 9-month score, then the mean score minus 10 points, then mean minus 20; scenario 5, a global score of 0 was imputed for patients who died within 9 months, all other patients (including those with disease progression) with a baseline global quality-of-life score but missing their 9-month global quality-of-life score were assigned the mean 9-month score. The rationale for scenarios 2–4 was that patients might have missed submitting their questionnaires due to illness, in which case a lower quality of life would be expected.

All analyses were done on an intention-to-treat basis with Stata version 15.0. The trial is registered on ClinicalTrials.gov, NCT01654146 and ISRCTN Registry, ISRCTN10356387.

### Role of the funding source

The funder of the study had no role in study design, data collection, data analysis, data interpretation, or writing of the report. SPB, ECJ, ARC, RK, and ADC had full access to the quality-of-life data in the study. The trial management group had final responsibility for the decision to submit for publication.

## Results

Between June 6, 2011, and Nov 28, 2014, 1566 patients were recruited into ICON8 (522 were included in group 1, 523 in group 2, and 521 in group 3). Baseline characteristics are described elsewhere.^4^ All patients were invited to participate in the quality-of-life study, and 1540 patients completed 17 515 quality-of-life questionnaires. 9 months after randomisation, quality-of-life data were expected from 1280 patients in follow-up without disease progression; 39 patients had died, 230 were alive after progression, and 17 had withdrawn or been lost to follow-up without progression. Baseline questionnaires were completed by 1438 (92%) of 1566 patients and 9-month questionnaires by 882 (69%) of 1280 patients (table 1). 828 (65%) of 1280 patients who could have contributed quality-of-life data at 9 months had both baseline and 9-month data and were included in cross-sectional analyses of primary and secondary outcomes.

**Table 1:** Completeness of quality-of-life data, by randomly allocated group and time

		**Group 1**		**Group 2**		**Group 3**	
		Patients	Quality of life	Patients	Quality of life	Patients	Quality of life
Baseline		522	475 (91%)	523	482 (92%)	521	481 (92%)
Chemotherapy							
	Start of cycle 3	513	428 (83%)	515	425 (83%)	508	403 (79%)
	Start of cycle 5	489	376 (77%)	502	357 (71%)	477	366 (77%)
Follow-up							
	6 weeks post treatment	456	259 (57%)	484	251 (52%)	455	249 (55%)
	9 months post treatment	410	288 (70%)	446	306 (69%)	424	288 (68%)

Data are n or n (%). Group 1=standard three-weekly carboplatin and paclitaxel. Group 2=three-weekly carboplatin and weekly dose-dense paclitaxel. Group 3=weekly carboplatin and weekly dose-dense paclitaxel.

**Figure 2:**
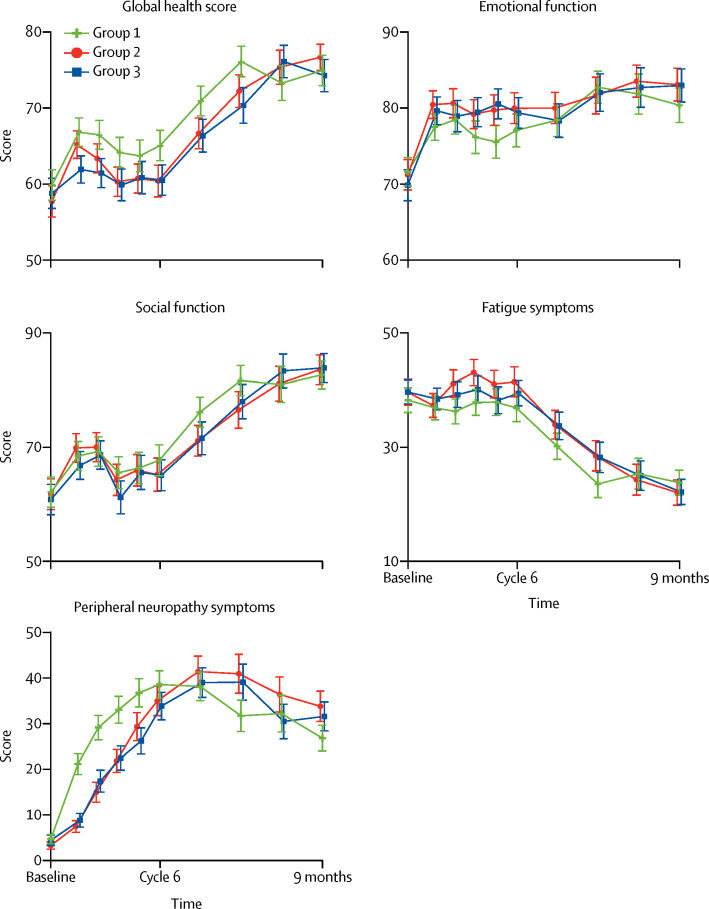
Primary and secondary quality-of-life endpoints For global health status and function scales (QLQ-C30 global health status, emotional function, and social function), higher scores indicate better function (improved quality of life) but for symptom scales (QLQ-C30 fatigue and QLQ-OV28 peripheral neuropathy), higher scores indicate greater symptoms (poorer quality of life). Error bars represent 95% CIs. The number of expected questionnaires and received questionnaires is reported in the appendix (p 5).

At the 9-month timepoint we found no significant difference in QLQ-C30 global health score between the three treatment groups with cross-sectional analysis (group 2 *vs* group 1, n=555, mean difference 2·3, 95% CI −0·4 to 4·9, p=0·094; group 3 *vs* group 1, n=522, mean difference −0·8, −3·8 to 2·2; p=0·61; table 2). We observed an improvement in mean global health score from baseline to 9 months post-randomisation across the study population (figure 2), although a fall in global health score was observed during chemotherapy in all three treatment groups. With longitudinal analysis, mean global health scores across the 9-month period were lower among patients in the weekly treatment groups, with a significant difference between group 3 and group 1 (group 2 *vs* group 1, 926 patients had global health score at baseline, and at least one score between baseline and 9 months, mean difference −1·8, 95% CI −3·6 to −0·1, p=0·043; group 3 *vs* group 1, n=915 patients had global health score at baseline, and at least one score between baseline and 9 months, mean difference −2·9, −4·7 to −1·1; p=0·0018; table 2). These results indicated poorer global health for patients who received weekly paclitaxel-containing treatment than for those who received 3-weekly chemotherapy but did not meet the threshold for clinical significance.

**Table 2:** QLQ-C30 global health score during first 9 months of treatment

		**Group 1 (n=410)**	**Group 2 (n=446)**	**Group 3 (n=424)**
Patients with global health score data at baseline and 9 months		264 (64%)	291 (65%)	258 (61%)
Global health score				
	Baseline	61·7 (22·1)	60·4 (23·0)	60·3 (22·8)
	9 months	74·8 (17·5)	76·9 (15·8)	73·9 (19·1)
Mean score over 9 months (SE)*		70·5 (0·9)	68·7 (1·0)	67·7 (1·0)
Difference in 9-month score *vs* group 1†		..	2·3 (−0·4 to 4·9); p=0·094	−0·8 (−3·8 to 2·2); p=0·61
Difference in mean score *vs* group 1		..	−1·8 (−3·6 to −0·1); p=0·043	−2·9 (−4·7 to −1·1); p=0·0018

Data are n (%), mean (SD), or mean (95% CI), unless otherwise indicated. Group 1=standard three-weekly carboplatin and paclitaxel. Group 2=three-weekly carboplatin and weekly dose-dense paclitaxel. Group 3=weekly carboplatin and weekly dose-dense paclitaxel.

* From area under the curve, calculated from mixed effects regression model.

† Adjusted for baseline.

We observed evidence of better emotional functioning (QLQ-C30) with weekly treatment (group 3) than with 3-weekly treatment (group 1) using cross-sectional analysis; however, it did not meet the threshold for clinical significance (table 3; figure 2). Longitudinal analysis revealed no significant differences in emotional functioning between study groups (table 3; figure 2). We found no difference in social functioning (QLQ-C30) between treatment groups using either cross-sectional or longitudinal analyses (table 3; figure 2). We observed no difference in fatigue (QLQ-C30) between groups by cross-sectional analysis; although fatigue scores were significantly different between the weekly (group 2) and 3-weekly (group 1) treatment groups by longitudinal analysis, they did not meet the threshold for clinical significance (table 3; figure 2). By cross-sectional analysis, peripheral neuropathy scores (QLQ-OV28) were statistically and clinically significantly different between group 2 and group 1; however, there was no difference by longitudinal analysis (table 3; figure 2). The timing of neuropathy differed between groups (figure 2).

**Table 3:** QLQ-C30 emotional function, social function, fatigue symptom, and QLQ-OV28 peripheral neuropathy symptom scores during first 9 months of treatment

		**Group 1 (n=410)**	**Group 2 (n=446)**	**Group 3 (n=424)**
**Emotional function**				
Patients with emotional function data at baseline and 9 months		265 (65%)	290 (65%)	261 (62%)
	Baseline*	73·1 (20·9)	74·8 (20·7)	70·5 (23·5)
	9 months	80·0 (20·6)	82·9 (19·6)	82·8 (19·6)
Mean score over 9 months (SE)†		79·0 (0·8)	80·6 (0·9)	80·5 (0·9)
Difference in 9-month score *vs* group 1‡		..	2·0 (−1·0 to 5·0); p=0·19	3·7 (0·5 to 6·8); p=0·024
Difference in mean score *vs* group 1		..	1·6 (−0·2 to 3·5); p=0·082	1·6 (−0·3 to 3·4); p=0·10
**Social function**				
Patients with social function data at baseline and 9 months		265 (65%)	290 (65%)	261 (62%)
	Baseline*	63·5 (30·5)	65·4 (30·1)	61·1 (31·3)
	9 months	82·8 (22·2)	83·7 (23·7)	84·1 (22·5)
Mean score over 9 months (SE)†		78·3 (1·2)	76·5 (1·3)	76·8 (1·3)
Difference in 9-month score *vs* group 1‡		..	0·4 (−3·4 to 4·1); p=0·85	1·7 (−1·9 to 5·3); p=0·36
Difference in mean score *vs* group 1		..	−1·8 (−4·3 to 0·7); p=0·15	−1·5 (−4·0 to 1·0); p=0·24
**Fatigue**				
Patients with fatigue data at baseline and 9 months		269 (66%)	292 (65%)	266 (63%)
	Baseline*	36·3 (25·4)	36·8 (24·9)	38·1 (24·8)
	9 months	23·7 (19·4)	22·1 (21·0)	22·5 (20·2)
Mean score over 9 months (SE)†		32·7 (1·0)	35·4 (1·1)	34·8 (1·1)
Difference in 9-month score *vs* group 1‡		..	−1·8 (−4·8 to 1·3); p=0·26	−1·8 (−4·9 to 1·3); p=0·25
Difference in mean score *vs* group 1		..	2·7 (0·6 to 4·8); p=0·011	2·1 (−0·1 to 4·2); p=0·057
**Peripheral neuropathy**				
Patients with peripheral neuropathy data at baseline and 9 months		260 (63%)	280 (63%)	260 (61%)
	Baseline*	5·1 (13·4)	2·4 (8·0)	4·1 (13·1)
	9 months	27·0 (25·7)	34·0 (31·0)	31·8 (28·3)
Mean score over 9 months (SE)†		32·9 (1·3)	31·8 (1·5)	31·2 (1·5)
Difference in 9-month score *vs* group 1‡		..	8·6 (3·8 to 13·3); p<0·0001	5·2 (0·5 to 9·8); p=0·028
Difference in mean score *vs* group 1		..	−1·2 (−4·2 to 1·8); p=0·45	−1·8 (−4·8 to 1·3); p=0·25

Data are n (%), mean (SD), or mean (95% CI) unless otherwise indicated. Group 1=standard three-weekly carboplatin and paclitaxel. Group 2=three-weekly carboplatin and weekly dose-dense paclitaxel. Group 3=weekly carboplatin and weekly dose-dense paclitaxel.

* Patients with quality-of-life data at baseline and 9 months.

† From area under the curve, calculated from mixed effects regression model.

‡ Adjusted for baseline.

Exploratory cross-sectional analyses of the other QLQ-C30 and QLQ-OV28 subscales showed that differences between the three randomised groups were generally small (appendix pp 5–6). We did not analyse data on sexual function since more than 80% of patients reported that they were sexually inactive at baseline.

A post-hoc analysis of peripheral neuropathy scores showed that, for patients who remained in follow-up without progression, high scores were still observed 18 months after randomisation for all treatment groups (appendix p 7). There was a statistically and clinically significant difference between group 2 and group 1 (n=298 with neuropathy score at baseline and 18 months, mean difference 10·7, 95% CI 4·2 to 17·2; p=0·0012) but not group 3 and group 1 (n=316 patients in group 1 or group 3 with neuropathy score at baseline and 18 months, 4·8, −0·9 to 10·4; p=0·096) by cross-sectional analysis. Longitudinal analysis did not show significant differences between the groups (group 2 *vs* group 1, n=898 patients with neuropathy score at baseline and at least one neuropathy score between baseline and 18 months, mean difference 2·5, 95% CI −1·2 to 6·1, p=0·18; group 3 *vs* group 1, n=901 patients with neuropathy score at baseline and at least one neuropathy score between baseline and 18 months, 2·5, −1·2 to 6·1, p=0·19).

Post-hoc analyses were done to investigate differences in quality of life related to timing of surgery (immediate primary surgery *vs* delayed primary surgery; appendix pp 8–10). Baseline characteristics by surgery timing are shown in the appendix (p 11). QLQ-C30 global health score at baseline was significantly lower among patients who had delayed surgery (n=714) than those who had immediate surgery (n=675) (mean 56·2 [SD 24·3] *vs* mean 61·7 [21·6]; p<0·0001). Baseline emotional function (delayed surgery, n=716, mean 68·6 [SD 23·6]; immediate surgery, n=673, mean 73·6 [21·5]; p<0·0001) and fatigue (delayed surgery, n=720, mean 42·3 [SD 27·0]; immediate surgery, n=679, mean 35·8 [22·9]; p<0·0001) were also significantly worse among patients who had delayed surgery, but we observed no difference in baseline scores for social function or peripheral neuropathy between patients with different surgery timings (data not shown). For patients who had immediate surgery, longitudinal analysis of the global health score over 9 months mirrored that in the overall study population (appendix pp 8–10). However, among patients who had delayed surgery the difference between treatment groups was smaller than it was in the overall study population (appendix pp 8–10).

We did a post-hoc analysis to assess concordance between self-reported peripheral neuropathy from the quality-of-life questionnaires and clinician-assessed peripheral neuropathy reported during the treatment period; there was good agreement between the measures (appendix p 12). Sensitivity analyses to assess the potential effect of missing data did not alter our interpretation of the data (appendix p 13).

## Discussion

The primary outcome of this study showed no difference between treatment groups in global health score at 9 months compared with baseline. However, longitudinal analysis revealed lower global health scores during chemotherapy for those in the weekly treatment groups than for those in the 3-weekly treatment group, which, although statistically significant, was of marginal clinical significance. These findings indicate that patients receiving weekly paclitaxel-containing chemotherapy had slower improvement in their overall quality of life during treatment itself, but recovered to a similar level to those receiving 3-weekly treatment at 9 months. Other secondary outcomes revealed slightly worse fatigue and more persistent peripheral neuropathy in the weekly paclitaxel-containing groups than in the 3-weekly group, although other quality-of-life subscores were similar between the study groups. Patients in the 3-weekly treatment group had an earlier onset of, but more rapid improvement in, peripheral neuropathy symptoms after completion of chemotherapy than did patients in the weekly treatment groups. By contrast, symptoms of peripheral neuropathy developed more gradually for patients in the weekly treatment groups compared with the 3-weekly treatment group but persisted beyond the end of treatment and into the follow-up period.

The observed differences in these quality-of-life subscores are probably due to paclitaxel exposure. As with the JGOG-3016 and GOG-0262 studies,^11^, ^12^ patients in group 2 and group 3 of ICON8 received up to 80 mg/m^2^ of paclitaxel per week, equivalent to 240 mg/m^2^ per cycle. In these three studies, peripheral neuropathy was worse in the weekly treatment groups than in the 3-weekly treatment group. By contrast, in the MITO-7 study,^13^ a lower weekly dose of 60 mg/m^2^ paclitaxel was administered in the weekly group (equivalent to 180 mg/m^2^ per cycle) and lower neuropathy scores were reported in the weekly group compared to those in the 3-weekly group (who received a similar per cycle dose of paclitaxel; appendix p 14). These findings suggest that peripheral neuropathy could be caused by cumulative exposure to paclitaxel rather than dosing intensity, although the exact mechanism of taxane neurotoxicity remains incompletely understood.

The large size of ICON8 allowed comparison between the quality-of-life trajectories of patients who had immediate primary surgery and delayed primary surgery. As the choice of upfront or delayed surgery was not randomly assigned, patients with more advanced-stage disease and poorer performance status were selected for upfront chemotherapy and delayed surgery, intended to reduce their anaesthetic risk and allow chemotherapy downstaging. Overall, patients who had delayed surgery had a larger incremental improvement in quality of life from randomisation to 9 months than did patients with immediate surgery, although having started from a lower point this result might have been expected. Their nadir quality-of-life score occurred at randomisation and improved during chemotherapy (with a slight fall at the time of interval surgery). The treatment break imposed by delayed surgery might also have lessened the cumulative effect of chemotherapy in these patients. By contrast, patients who had immediate surgery entered ICON8 having recovered from surgery and with better quality of life; although they had a quality-of-life nadir following chemotherapy (at around 18 weeks), their quality of life recovered to the same level as those in the delayed surgery group at 9 months. In patients who had immediate surgery, treatment-related fatigue and global quality-of-life were significantly (and clinically) worse in the weekly paclitaxel-containing groups than in the 3-weekly treatment group.

To address the immediate and long-term effect of treatment on quality of life in ICON8, we chose an intensive method of quality-of-life data collection during treatment and in post-treatment follow-up. Thus, questionnaires were completed at every treatment cycle rather than at the beginning, middle, and end of treatment. We recommend this close method of tracking of quality of life, along with longitudinal rather than cross-sectional methods of analysis, to more accurately reflect changes over time. Alternatively, standardised, continuous patient-reported outcome measures can be implemented to provide more reliable monitoring of the longer-term toxicities that affect patient wellbeing.^12^ Because of the differences between non-randomised patients undergoing immediate or delayed surgery, we recommend that these two surgical groups are analysed separately in future quality-of-life studies.

To our knowledge, ICON8 provides the largest and most detailed quality-of-life dataset of any neoadjuvant ovarian cancer trial. A limitation of the study is the loss of quality-of-life data due to patients who did not complete or return their questionnaires, which potentially introduced bias against those with more severe symptoms who might have been less able or willing to comply. However, we did not observe a difference in data return between the treatment groups, indicating this was unlikely to be a consequence of a specific regimen. Questionnaire compliance was favourable compared with other similar studies; baseline quality-of-life questionnaires were completed by 92% of patients in ICON8 compared with 63·9% and 75% in the JGOG-3016 and MITO-7 studies, respectively. Although we noted numerous changes in quality-of-life subscores across the groups, many did not reach clinical significance. We selected a change in score of 5 points as being clinically significant, based on studies of snapshot rather than longitudinal quality-of-life comparisons. Hence, in this context, the cutoff is exploratory and possibly too stringent for defining the clinical significance of the quality-of-life subscales. There might also be clinical reasons for the lack of clinical significance observed, such as different causalities causing similar symptoms. For example, patients with bulky, unresected tumours at baseline might report ongoing fatigue that is replaced by drug-related fatigue once chemotherapy is underway. Additionally, response shift is a limitation of quality-of-life studies, whereby patients adapt to and therefore underreport symptoms over time.^14^

As the main ICON8 study revealed no progression-free survival advantage for weekly paclitaxel-containing regimens, our quality-of-life data do not support its use in favour of standard 3-weekly carboplatin and paclitaxel after upfront surgery in ovarian cancer. However, our finding of no quality of life detriment between the groups among patients who had delayed surgery suggests that, in those unsuitable for upfront surgery, 3-weekly and weekly schedules might be equivocal. In patients who have delayed surgery, who are likely to be in poorer health, the weekly scheduling of both carboplatin and paclitaxel allows more careful dosing modulation and symptom management than 3-weekly dosing, and is equivalent to 3-weekly dosing in terms of its effect on global quality of life.

In conclusion, for patients undergoing upfront surgery, weekly paclitaxel-containing chemotherapy should not replace 3-weekly carboplatin and paclitaxel as the standard of care for newly diagnosed ovarian cancer, since it neither improves progression-free survival nor is associated with improved quality of life.

## Data sharing

Data will be shared according to the Medical Research Council Clinical Trial Unit controlled access approach, based on the following principles: no data should be released that would compromise an ongoing trial or study; there must be a strong scientific or other legitimate rationale for the data to be used for the requested purpose; investigators who have invested time and effort into developing a trial or study should have a period of exclusivity in which to pursue their aims with the data, before key trial data are made available to other researchers; the resources required to process requests should not be underestimated, particularly successful requests that lead to preparing data for release, therefore, adequate resources must be available to comply in a timely manner or at all, and the scientific aims of the study must justify the use of such resources; and data exchange complies with Information Governance and Data Security Policies in all of the relevant countries. Researchers wishing to access ICON8 data should contact mrcctu.icon8and8b@ucl.ac.uk.
