# Supplementary material for: Weekly platinum-based chemotherapy versus 3-weekly platinum-based chemotherapy for newly diagnosed ovarian cancer (ICON8): quality-of-life results of a phase 3, randomised, controlled trial
Source: Lancet Oncol. 2020 Jul;21(7):969–77. doi: 10.1016/S1470-2045(20)30218-7 (PMC7327508; doi:10.1016/S1470-2045(20)30218-7)
Supplement: Supplementary appendix [file mmc1.pdf]

THE LANCET
Oncology

Supplementary appendix

This appendix formed part of the original submission and has been peer reviewed. We post it as supplied by the authors.

Supplement to: Blagden SP, Cook AD, Poole C, et al. Weekly platinum-based chemotherapy versus 3-weekly platinum-based chemotherapy for newly diagnosed ovarian cancer (ICON8): quality-of-life results of a phase 3, randomised, controlled trial. *Lancet Oncol* 2020; **21:** 969–77.

Appendix Table 1: List of all Recruiting Sites and PIs

Site name	Country	Accrual	Principal Investigator
Christie Hospital	UK	105	Andrew Clamp
Hammersmith Hospital	UK	70	Hani Gabra
University College London Hospital	UK	51	Jonathan Ledermann
Clatterbridge Centre for Oncology	UK	57	Rosemary Lord
Freeman Hospital	UK	45	Graham Dark
Addenbooke's Hospital	UK	43	Helena Earl
University Hospital Coventry and Warwickshire	UK	42	Chris Poole
Mount Vernon Hospital	UK	41	Marcia Hall
Instituto Nacional de Cancerologia	Mexico	39	Dolores Gallardo
Beatson West of Scotland Cancer Centre	UK	38	Ros Glasspool
St Bartholomew's Hospital (London)	UK	36	Michelle Lockley
City Hospital (Birmingham)	UK	33	Sarah Williams
Velindre Hospital	UK	33	Rachel Jones
Royal Surrey County Hospital	UK	31	Sharadah Essapen
Bristol Haemotology & Oncology Centre	UK	28	Axel Walther
Seoul National University Hospital	S. Korea	27	Jae Weon Kim
Royal Devon and Exeter Hospital	UK	26	Kate Scatchard
Maidstone Hospital	UK	25	Jeff Summers
Royal Shrewsbury Hospital	UK	25	Saif Awwad
Nottingham University Hospitals	UK	24	Stephen Chan
Royal Derby Hospital	UK	23	Mojca Persic
Broomfield Hospital	UK	22	Saad Tahir
James Cook University Hospital	UK	21	Louise Li
Royal Marsden Hospital (Sutton)	UK	21	Susana Banerjee
Guy's and St Thomas' Hospital (London)	UK	19	Ana Montes
Royal Marsden Hospital (London)	UK	19	Susana Banerjee
Cheltenham General Hospital	UK	18	Audrey Cook
St Helens Hospital	UK	18	Rosemary Lord
St James University Hospital (Leeds)	UK	18	Timothy Perren
North Devon District Hospital	UK	17	Kate Scatchard
Royal United Hospital, Bath	UK	15	Rebecca Bowen
University Hospital of North Staffordshire	UK	15	Rajanee Bhana
Weston Park Hospital	UK	14	Simon Pledge
Wexham Park	UK	14	Marcia Hall
Great Western Hospital	UK	13	Omar Khan
Queen Elizabeth The Queen Mother Hospital	UK	13	Justin Waters
St George's Hospital (London)	UK	13	Fiona Lofts
Warwick Hospital	UK	13	Nawaz Walji
Mater Misericordiae University Hospital	R. of Ireland	12	John McCaffery
Musgrove Park Hospital	UK	12	Clare Barlow
Singleton Hospital	UK	12	Gianfilippo Bertelli
St John of God Hospital Subiaco	AUS	12	Andrew Dean
Aberdeen Royal Infirmary	UK	11	Trevor McGoldrick

Churchill Hospital	UK	11	Shibani Nicum
Glan Clwyd Hospital	UK	11	Anna Mullard
Royal Cornwall Hospital	UK	11	Nigel Bailey
Southend University Hospital	UK	11	Naveed Sarwar
Dorset County Hospital	UK	10	Maxine Flubacher
Northampton General Hospital	UK	10	Roshan Agarwal
Peterborough City Hospital	UK	10	Sarah Ayers
Queen's Hospital (Romford)	UK	10	Mary Quigley
Royal Berkshire Hospital	UK	10	Madhumita Bhattacharyya
Airedale General Hospital	UK	9	Michael Crawford
Huddersfield Royal Infirmary & Calderdale Royal Hospital	UK	9	Uschi Hofmann
Liverpool Women's Hospital	UK	9	Rosemary Lord
Ninewells Hospital	UK	9	Michelle Ferguson
Westmead Hospital	AUS	9	Alison Brand
Belfast City Hospital	UK	8	Sarah Mckenna
Norfolk and Norwich University Hospital	UK	8	Daniel Epurescu
Royal Lancaster Infirmary	UK	8	Sarah Moon
Royal Preston Hospital	UK	8	Andrew Hindley
Sir Charles Gairdner Hospital	AUS	8	Tarek Meniawy
Staffordshire General Hospital	UK	8	Rajanee Bhana
Yeovil District Hospital	UK	8	Clare Barlow
Auckland City Hospital	NZ	7	Kathryn Chrystal
Calvary Mater Newcastle	AUS	7	Janine Lombard
Hinchingbrooke Hospital	UK	7	Li Tee Tan
Leicester Royal Infirmary	UK	7	David Peel
Queen Alexandra Hospital	UK	7	Chit Cheng Yeoh
Royal Sussex County Hospital	UK	7	Rebecca Herbertson
Bedford Hospital	UK	6	Sarah Smith
Cumberland Infirmary	UK	6	Syed Asghar
Doncaster Royal Infirmary	UK	6	Simon Pledge
George Eliot Hospital	UK	6	Mark Hocking
Torbay District General Hospital	UK	6	Nangi Lo
Victoria Hospital (Blackpool)	UK	6	Andrew Hindley
Weston General Hospital	UK	6	Waheeda Owadally
Queen's Hospital (Burton)	UK	5	Lalith Seneviratne
Waterford Regional Hospital	R. of Ireland	5	Paula Calvert
Withybush Hospital	UK	5	David Davies
ASAN Medical Center	S. Korea	4	Joo-hyun Nam
Castle Hill Hospital	UK	4	Georgios Bozas
Centro Oncologico Estatal	Mexico	4	Eva Maria Gomez
New Cross Hospital	UK	4	Rozenn Allerton
Royal Women's Hospital	AUS	4	Sumitra Ananda
Southampton General Hospital	UK	4	Vicky Mcfarlane
Worthing Hospital	UK	4	Rebecca Herbertson
Ysbyty Gwynedd	UK	4	Nick Stuart
Beaumont Hospital	R. of Ireland	3	Patrick Morris
Border Medical Oncology	AUS	3	Christopher Steer

Diana Princess of Wales	UK	3	Mohammad Butt
Hereford County Hospital	UK	3	Audrey Cook
Ipswich Hospital	UK	3	Jamie Morgan
Kettering General Hospital	UK	3	Choi Mak
Mater Private Hospital	R. of Ireland	3	David Gallagher
York District Hospital	UK	3	Angela Darby
Bankstown Hospital	AUS	2	Sandra Harvey
Bradford Royal Infirmary	UK	2	Sue Cheeseman
Canberra Hospital	AUS	2	Sayed Ali
Mercy Hospital for Women	AUS	2	Linda Mileshkin
Peninsula Oncology Centre	AUS	2	Yoland Antill
Poole Hospital	UK	2	Richard Osborne
Prince of Wales Hospital	AUS	2	Michael Friedlander
Royal Blackburn Hospital	UK	2	Martin Hogg
Royal Brisbane & Women's Hospital	AUS	2	Alison Hadley
St George's Hospital (ANZ)	AUS	2	Chee Lee
Townsville Hospital	AUS	2	Judith Page
Chris O'Brien Lifehouse	AUS	1	Philip Beale
Christchurch Hospital	NZ	1	Michelle Vaughan
Gangnam Sevrance Hospital	S. Korea	1	Jae Hoon Kim
James Paget University Hospital	UK	1	Debashis Biswas
Lister Hospital	UK	1	Marcia Hall
Manor Hospital	UK	1	Indrajit Fernando
Monash Health	AUS	1	Geraldine Goss
Queen Elizabeth Hospital (Kings Lynn)	UK	1	Margaret Daly
St James's (Dublin)	R. of Ireland	1	Dearbhaile O'Donnell
Western Hospital	AUS	1	Sumitra Ananda

Appendix Figure 1: ICON8 trial schema

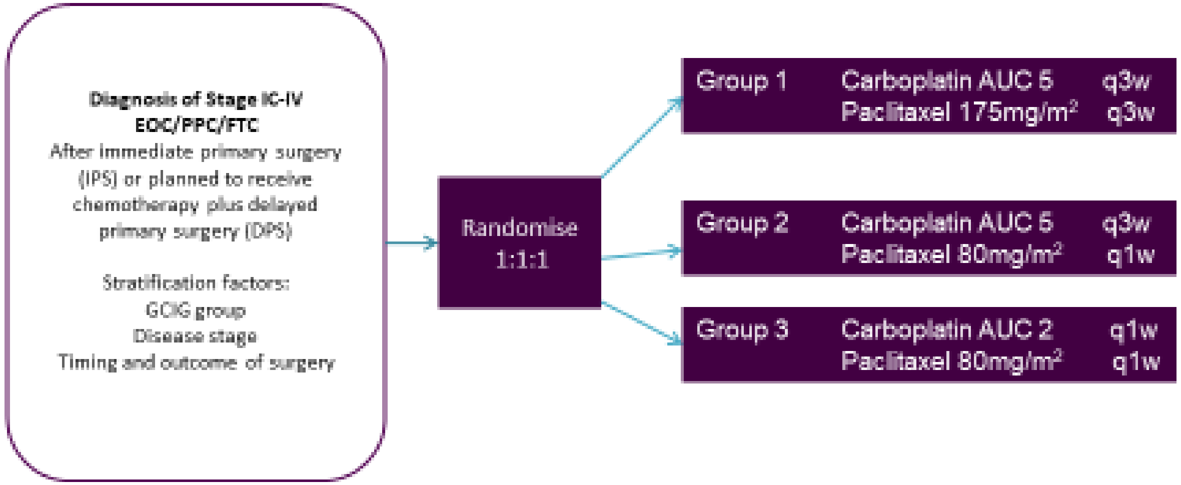

Appendix Table 2. Completeness of data presented in figure 2.

Global health, emotional and social function, fatigue and peripheral neuropathy. By treatment group and time point.

**GROUP 1**	Expected	Received									
		Global health score		Emotional funct. score		Social func. score		Fatigue score		Periph. neur. score	
	N	N	(%)	N	(%)	N	(%)	N	(%)	N	(%)
Baseline	522	474	(91)	473	(91)	473	(91)	475	(91)	459	(88)
Cycle 2	516	439	(85)	439	(85)	438	(85)	443	(86)	440	(85)
Cycle 3	513	425	(83)	425	(83)	426	(83)	428	(83)	424	(83)
Cycle 4	500	383	(77)	383	(77)	383	(77)	384	(77)	381	(76)
Cycle 5	496	372	(75)	375	(76)	374	(75)	378	(76)	373	(75)
Cycle 6	489	374	(76)	376	(77)	376	(77)	376	(77)	371	(76)
End of trt	474	346	(73)	346	(73)	347	(73)	348	(73)	346	(73)
6 weeks	456	258	(57)	259	(57)	259	(57)	259	(57)	256	(56)
12 weeks	433	192	(44)	193	(45)	193	(45)	195	(45)	195	(45)
9 months	410	284	(69)	286	(70)	286	(70)	288	(70)	285	(70)

**GROUP 2**	Expected	Received									
		Global health score		Emotional func. score		Social func. score		Fatigue score		Periph. neur. score	
	N	N	(%)	N	(%)	N	(%)	N	(%)	N	(%)
Baseline	523	479	(92)	478	(91)	477	(91)	481	(92)	464	(89)
Cycle 2	518	451	(87)	451	(87)	450	(87)	453	(87)	451	(87)
Cycle 3	515	424	(82)	424	(82)	424	(82)	425	(83)	421	(82)
Cycle 4	506	395	(78)	395	(78)	395	(78)	396	(78)	393	(78)
Cycle 5	504	359	(71)	360	(71)	360	(71)	362	(72)	361	(72)
Cycle 6	502	354	(71)	354	(71)	354	(71)	355	(71)	355	(71)
End of trt	493	357	(72)	359	(73)	359	(73)	360	(73)	355	(72)
6 weeks	484	251	(52)	251	(52)	251	(52)	251	(52)	244	(50)
12 weeks	468	235	(50)	235	(50)	235	(50)	237	(51)	235	(50)
9 months	446	306	(69)	306	(69)	306	(69)	306	(69)	304	(68)

**GROUP 3**	Expected	Received									
		Global health score		Emotional func. score		Social func. score		Fatigue score		Periph. neur. score	
	N	N	(%)	N	(%)	N	(%)	N	(%)	N	(%)
Baseline	521	474	(91)	476	(91)	476	(91)	481	(92)	474	(91)
Cycle 2	514	450	(88)	451	(88)	451	(88)	452	(88)	446	(87)
Cycle 3	508	398	(78)	399	(79)	398	(78)	402	(79)	400	(79)
Cycle 4	482	359	(74)	360	(75)	360	(75)	361	(75)	357	(74)
Cycle 5	480	324	(68)	325	(68)	325	(68)	327	(68)	325	(68)

Cycle 6	477	365	(77)	365	(77)	365	(77)	366	(77)	362	(76)
End of trt	465	332	(71)	332	(71)	332	(71)	332	(71)	329	(71)
6 weeks	455	248	(55)	248	(55)	248	(55)	249	(55)	247	(54)
12 weeks	439	225	(51)	226	(51)	226	(51)	227	(52)	225	(51)
9 months	424	284	(67)	286	(67)	286	(67)	287	(68)	285	(67)

Appendix Table 3. Exploratory outcomes, other functional scores and symptom scores

	Baseline						9 months					
	Group 1		Group 2		Group 3		Group 1		Group 2		Group 3	
	mean	(sd)	mean	(sd)	mean	(sd)	mean	(sd)	mean	(sd)	mean	(sd)
**Function score**												
Physical	76.5	(20.3)	75.7	(21.6)	74.3	(20.6)	86.8	(15.1)	85.5	(17.3)	84.8	(17.9)
Role	57.8	(33.7)	58.9	(33.8)	55.1	(32.8)	81.0	(21.1)	80.7	(25.8)	81.0	(23.4)
Cognitive	84.8	(19.3)	84.0	(20.0)	81.8	(22.5)	83.7	(19.3)	84.3	(18.9)	85.1	(18.8)
**Symptom score**												
Nausea	9.9	(20.0)	10.3	(20.7)	11.6	(18.9)	3.4	(9.2)	3.7	(11.8)	3.0	(8.2)
Pain	28.9	(25.7)	29.3	(27.1)	29.7	(27.7)	16.2	(21.4)	15.5	(22.7)	15.9	(22.5)
Dyspnoea	14.2	(23.4)	14.8	(23.2)	17.4	(26.5)	12.7	(20.6)	12.3	(21.0)	13.7	(21.9)
Insomnia	35.6	(31.4)	36.9	(30.9)	36.5	(32.3)	24.6	(28.2)	24.8	(27.2)	23.5	(27.8)
Appetite loss	24.1	(29.2)	25.9	(31.1)	29.6	(32.3)	6.2	(16.1)	5.6	(16.0)	6.6	(17.4)
Constipation	24.8	(28.7)	21.5	(29.5)	24.7	(31.0)	11.8	(21.5)	8.6	(18.7)	9.4	(19.1)
Diarrhoea	10.4	(21.6)	9.1	(19.1)	11.8	(23.0)	4.6	(12.7)	5.3	(15.2)	4.4	(12.7)
Financial difficulty	16.6	(28.0)	16.0	(25.9)	18.6	(30.7)	11.6	(23.8)	12.4	(23.8)	13.1	(25.6)
Abdominal	31.3	(24.1)	33.2	(25.6)	33.5	(24.1)	13.0	(13.8)	12.8	(16.4)	12.5	(14.1)
Hormonal	19.3	(24.6)	19.2	(27.3)	23.3	(28.6)	20.4	(27.1)	21.7	(29.6)	22.1	(28.1)
Body image	22.5	(25.4)	25.3	(29.0)	26.6	(28.7)	25.6	(26.1)	24.0	(26.0)	24.0	(26.1)
Attitude to disease	48.2	(27.1)	45.6	(26.2)	47.9	(28.1)	33.8	(24.8)	29.0	(25.3)	29.2	(24.6)
Chemotherapy	13.8	(14.7)	14.3	(14.7)	14.1	(14.0)	17.1	(14.3)	16.6	(15.3)	19.5	(16.5)
Other symptoms	11.8	(15.9)	10.9	(13.3)	11.8	(15.7)	10.3	(17.5)	11.2	(17.6)	11.8	(18.3)

Appendix Table 4. Secondary outcome, global health score during first 18 months of treatment.

	Group 1	Group 2		Group 3	
Patients with global health score at baseline and 18 mths	n=150	n=155		n=170	
Global score, mean(sd)					
Baseline	64.1 (22.6)	62.7 (22.2)		62.9 (22.4)	
18 months	78.7 (16.4)	75.7 (19.6)		74.0 (19.5)	
Mean (SE) score over 18 months[a]	70.1 (1.0)	68.8 (1.0)		68.2 (1.0)	
Difference in 18 month score[b], mean (95%CI)					
Cross-sectional analysis		Group 2 vs 1	p	Group 3 vs 1	p
		-2.8 (-6.7,1.0)	0.15	-4.3 (-8.1,-0.5)	0.027
Difference in mean score, (95%CI)					
Longitudinal analysis		Group 2 vs 1	p	Group 3 vs 1	p
		-1.4 (-3.4,-0.7)	0.19	-2.0 (-4.0,-0.6)	0.057

a. from area under the curve, calculated from mixed effects regression model

b. adjusted for baseline

Appendix Figure 2: Peripheral neuropathy, by treatment group from baseline to 18 months

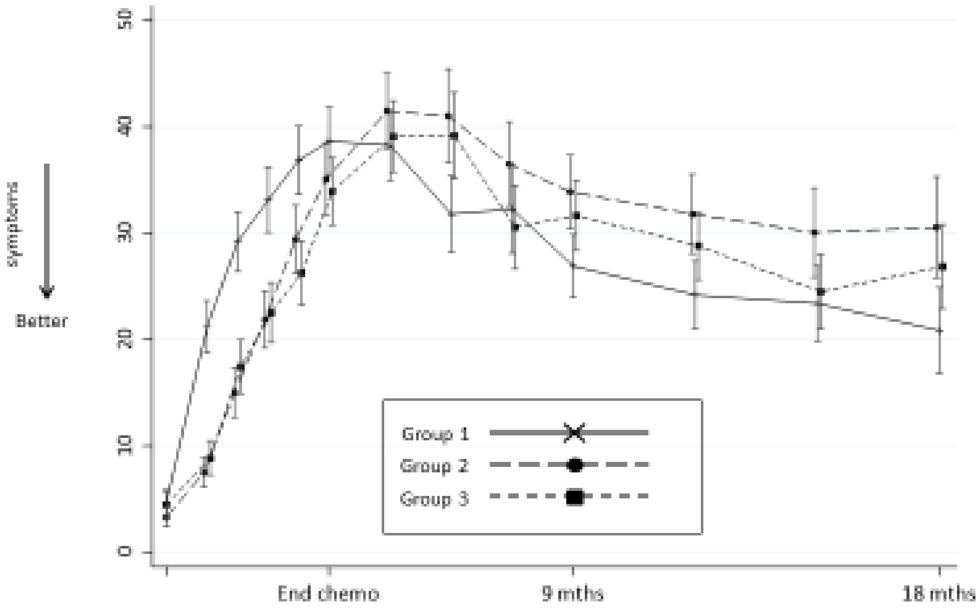

	Group 1	Group 2	Group 3
Peripheral neuropathy score at baseline and at 18 months	N=147	N=151	N=169
Baseline score<br>18 month score<br>Difference in 18 month score vs group 1	3.2 (9.0)<br>20.4 (25.0)	2.2 (7.3)<br>30.7 (31.4)<br>10.7 (4.2 to 17.2)<br>p=0.0012	4.9 (12.2)<br>26.4 (26.8)<br>4.8 (-0.9 to 10.4)<br>p=0.096
Peripheral neuropathy score at baseline and at least one score during follow-up to 18 months	N=446	N=452	N=455
Mean score over 18 months (SE)<br>Difference in mean score vs group 1	27.9 (1.6)	30.4 (1.7)<br>2.5 (-1.2 to 6.1)<br>p=0.18	30.4 (1.7)<br>2.5 (-1.2 to 6.1)<br>p=0.19

Appendix Table 5. Primary and secondary outcomes, by timing of surgery. Results of cross-sectional and longitudinal analysis.

Cross-sectional analysis	Mean 9 month score						IPS				DPS			
	IPS			DPS			Group 2 v 1		Group 3 v 1		Group 2 v 1		Group 3 v 1	
	Group 1	Group 2	Group 3	Group 1	Group 2	Group 3	diff	p	diff	p	diff	p	diff	p
Global score	77.2	77.6	72.2	72.3	76.3	76.1	0.4	0.74	-5.0	0.01	4.1	0.05	3.8	0.11
Emotional function	82.6	83.6	81.4	77.2	83.0	84.0	1.1	0.87	-1.2	0.47	5.7	0.06	6.8	0.02
Social function	85.4	85.2	84.2	80.5	82.8	83.6	-0.2	0.70	-1.2	0.87	2.3	0.50	3.1	0.25
Fatigue symptoms	20.9	22.0	23.4	26.8	22.4	21.7	1.2	0.69	2.6	0.44	-4.4	0.07	-5.1	0.02
Neuropathy symptoms	25.3	40.2	31.5	28.3	28.1	31.4	14.9	<0.01	6.2	0.05	-0.3	0.60	3.1	0.30
Longitudinal analysis	Mean score						IPS				DPS			
	IPS			DPS			Group 2 v 1		Group 3 v 1		Group 2 v 1		Group 3 v 1	
	Group 1	Group 2	Group 3	Group 1	Group 2	Group 3	diff	p	diff	p	diff	p	diff	p
Global score	73.0	67.8	68.2	67.4	68.6	66.5	-5.2	<0.01	-4.8	<0.01	1.3	0.36	-0.9	0.55
Emotional function	81.3	81.9	81.8	78.6	81.3	80.9	0.5	0.65	0.5	0.66	2.8	0.06	2.4	0.12
Social function	78.3	73.5	76.1	71.2	72.4	70.7	-4.9	<0.01	-2.2	0.17	1.2	0.53	-0.5	0.80
Fatigue symptoms	30.4	36.2	34.2	36.2	35.9	36.5	5.9	<0.01	3.8	0.01	-0.4	0.83	0.3	0.86
Neuropathy symptoms	32.2	35.2	30.2	32.1	26.5	30.0	3.0	0.16	-2.0	0.34	-5.6	0.01	-2.1	0.36

Appendix Figure 3: QOL by IPS/DPS and treatment group

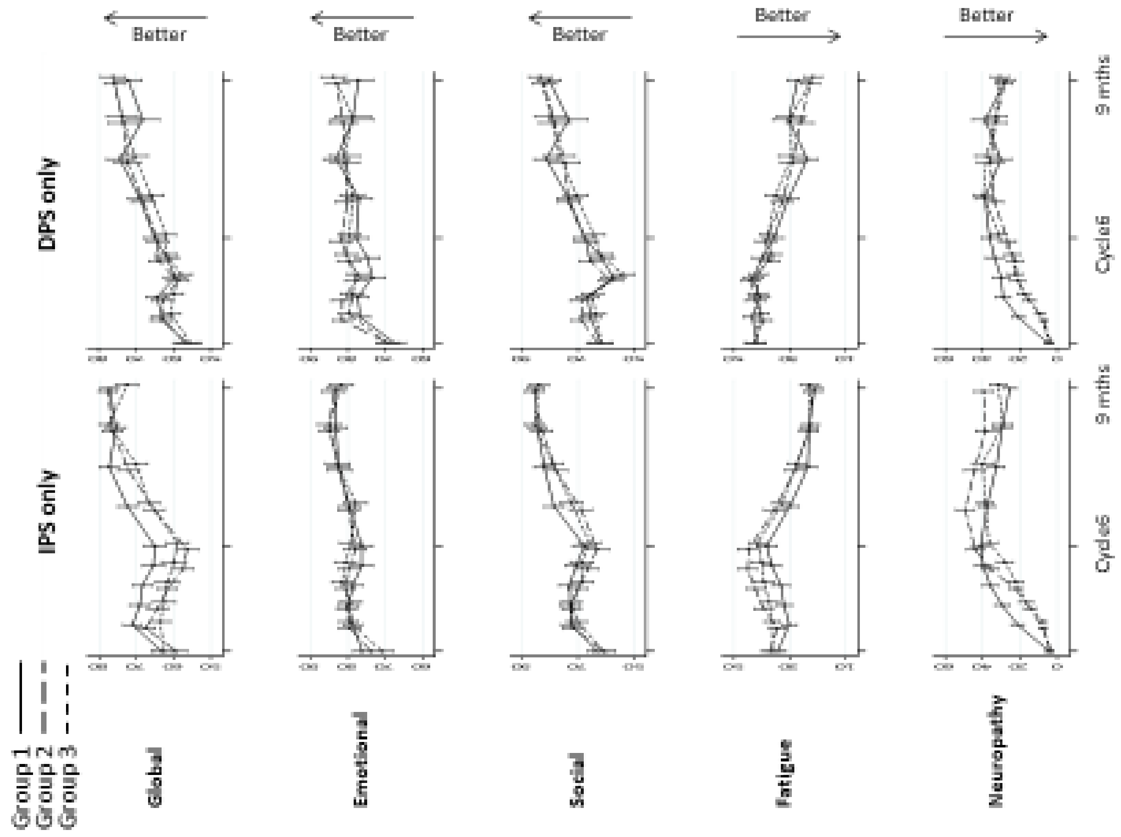

Appendix Figure 4: Primary and Secondary QOL endpoints by surgical timing

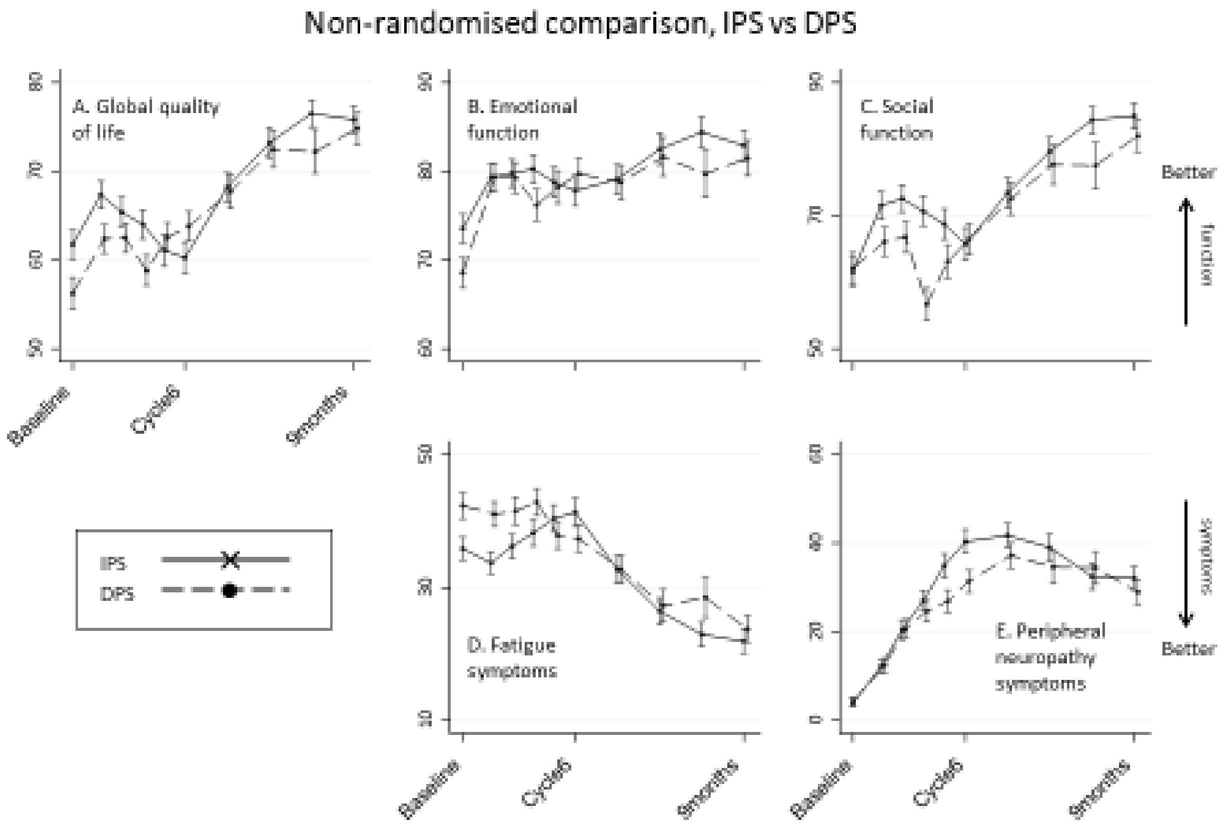

Appendix Table 6: Baseline Characteristics by Timing of Surgery

	**Immediate Surgery (N=746)**	**Delayed Surgery or Inoperable (N=820)**
Age (years)	60 (52-66)*	64 (56-69)*
Origin		
Ovary (Epithelial)	659 (88%)	618 (76%)
Fallopian tube	63 (8%)	9 (1%)
Primary Peritoneal	23 (3%)	189 (23%)
Missing	*1*	*4*
Histological subtype		
Serous high grade	468 (63%)	605 (74%)
Serous low grade	14 (2%)	14 (2%)
Serous (no grade specified)	0	25 (3%)
Clear cell	94 (13%)	13 (2%)
Endometroid	60 (8%)	7 (1%)
Carcinosarcoma	10 (1%)	2 (<1%)
Mixed/other	100 (13%)	154 (19%)
FIGO Stage		
IC/IIA	160 (21%)	4 (<1%)
IIB/IIC	123 (16%)	8 (1%)
IIIA/IIIB	117 (16%)	35 (4%)
IIIC	288 (39%)	523 (64%)
IV	58 (8%)	250 (30%)
ECOG performance status		
0	405 (55%)	326 (40%)
1	313 (42%)	400 (49%)
2	25 (3%)	91 (11%)
Missing	*3*	*3*

*median (IQR)

Appendix Figure 5: Clinician-reported peripheral neuropathy adverse events and patient reported neuropathy on Quality of Life questionnaires

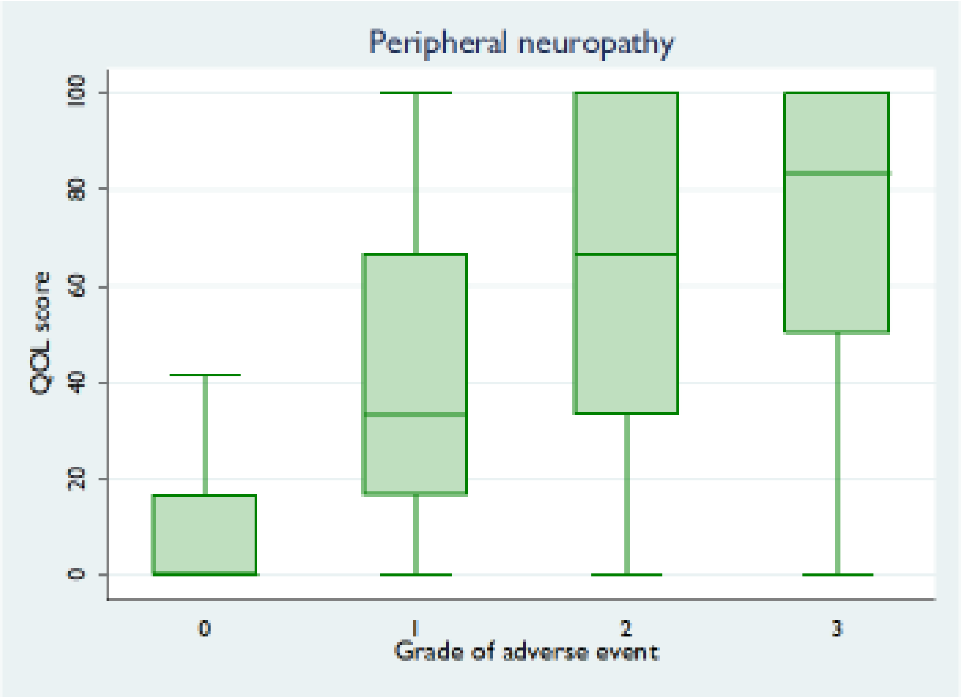

Appendix Figure 6: Sensitivity analysis of difference in Global QOL

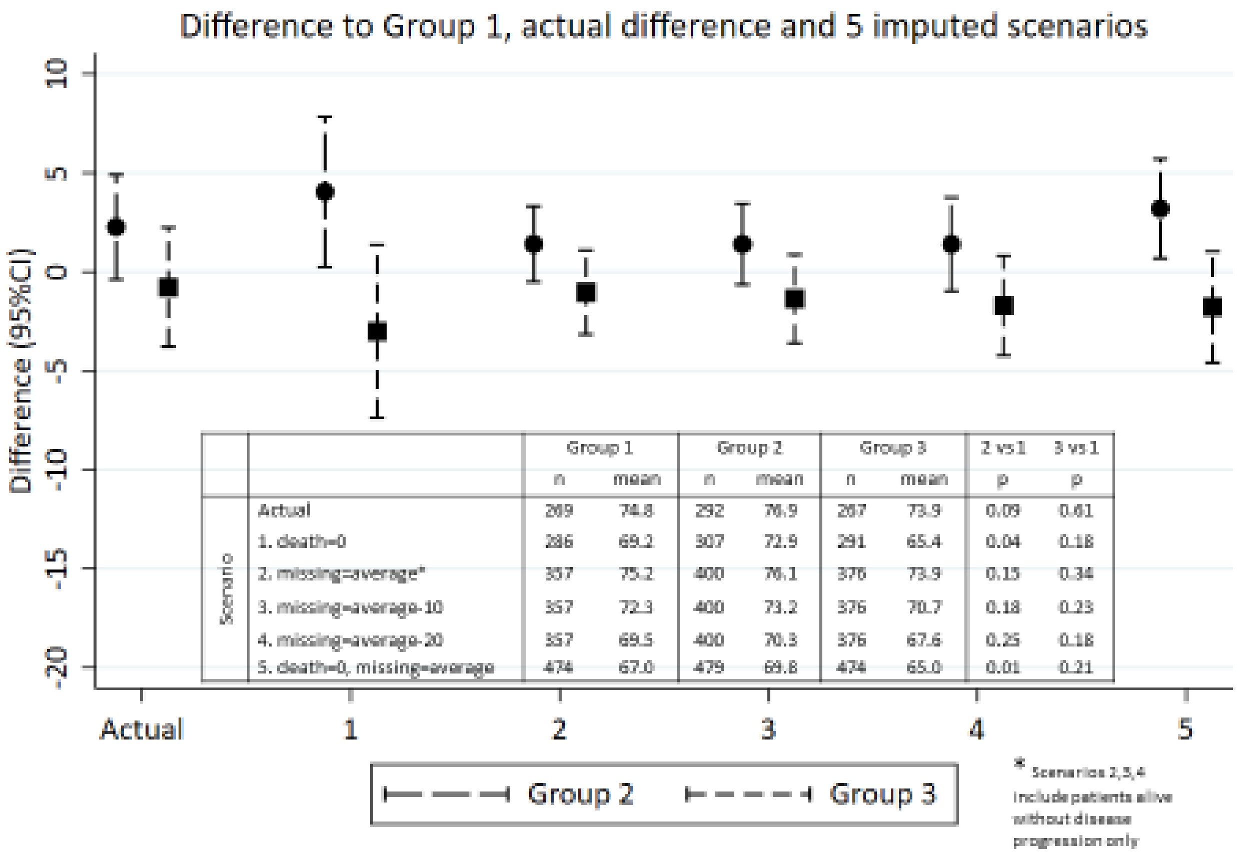

		Group 1		Group 2		Group 3		2 vs 1	3 vs 1
		n	mean	n	mean	n	mean	p	p
Scenario	Actual	269	74.8	292	76.9	267	73.9	0.09	0.61
	1. death=0	286	69.2	307	72.9	291	65.4	0.04	0.18
	2. missing=average*	357	75.2	400	76.1	376	73.9	0.15	0.34
	3. missing=average-10	357	72.3	400	73.2	376	70.7	0.18	0.23
	4. missing=average-20	357	69.5	400	70.3	376	67.6	0.25	0.18
	5. death=0, missing=average	474	67.0	479	69.8	474	65.0	0.01	0.21

Appendix Table 7: Comparison with other weekly treatment trials

	Treatment			PFS HR(95% CI)		Quality of Life		
	A. Carb+Pac 3 weekly	B. Carb 3 weekly + Pac weekly	C. Carb+Pac weekly	B vs A	C vs A	Global QOL with weekly treatment	Neuropathy with weekly treatment	Change over time
**ICON8**	522	523	521	0.9 (0.77,1.05)	0.93 (0.78,1.08)	Worse[a]	Worse[b]	Improved
**JGOG 3016**	319	312	-	0.76 (0.62,0.91)	-	None[c]	Worse[c]	No change
**GOG-0262**	346	346	-	0.89 (0.74,1.06)	-	Worse[d]	Worse[d]	Improved
**MITO-7**	404	-	406	-	0.96 (0.8,1.16)	Better[e]	Better[e]	No change[e]

a. Longitudinal analysis from baseline to 9 months, B vs. A and C vs. A.
b. Cross-sectional analysis at 9 months, B vs. A and C vs. A.
c. Longitudinal analysis from baseline to 12 months.
d. Longitudinal analysis from baseline to 12+ months.
e. Baseline to 9 weeks only.
